# Mutant p53-R273H mediates cancer cell survival and anoikis resistance through AKT-dependent suppression of BCL2-modifying factor (BMF)

**DOI:** 10.1038/cddis.2015.191

**Published:** 2015-07-16

**Authors:** B S Tan, K H Tiong, H L Choo, F Fei-Lei Chung, L-W Hii, S H Tan, I KS Yap, S Pani, N TW Khor, S F Wong, R Rosli, S-K Cheong, C-O Leong

**Affiliations:** 1 School of Postgraduate Studies, International Medical University, Bukit Jalil, Kuala Lumpur, Malaysia; 2 Center for Cancer and Stem Cell Research, International Medical University, Bukit Jalil, Kuala Lumpur, Malaysia; 3 Oral Cancer Research and Co-ordinating Center (OCRCC), Faculty of Dentistry, University of Malaya, Kuala Lumpur, Malaysia; 4 Cancer Research Initiatives Foundation, Sime Darby Medical Centre, Subang Jaya, Malaysia; 5 School of Pharmacy, International Medical University, Bukit Jalil, Kuala Lumpur, Malaysia; 6 ANU Medical School, Canberra Hospital Campus, The Canberra Hospital Building 4, Garran, Australia; 7 School of Medicine, Faculty of Medical and Health Sciences, The University of Auckland, Auckland, New Zealand; 8 School of Medicine, International Medical University, Bukit Jalil, Kuala Lumpur, Malaysia; 9 UPM-MAKNA Cancer Research Laboratory, Institute of Bioscience, Universiti Putra Malaysia, 43400 UPM Serdang, Selangor, Malaysia; 10 Faculty of Medicine and Health Sciences, University Tunku Abdul Rahman, Bandar Sungai Long, Selangor, Malaysia

## Abstract

p53 is the most frequently mutated tumor-suppressor gene in human cancers. Unlike other tumor-suppressor genes, p53 mutations mainly occur as missense mutations within the DNA-binding domain, leading to the expression of full-length mutant p53 protein. Mutant p53 proteins not only lose their tumor-suppressor function, but may also gain new oncogenic functions and promote tumorigenesis. Here, we showed that silencing of endogenous p53-R273H contact mutant, but not p53-R175H conformational mutant, reduced AKT phosphorylation, induced BCL2-modifying factor (BMF) expression, sensitized BIM dissociation from BCL-X_L_ and induced mitochondria-dependent apoptosis in cancer cells. Importantly, cancer cells harboring endogenous p53-R273H mutant were also found to be inherently resistant to anoikis and lack BMF induction following culture in suspension. Underlying these activities is the ability of p53-R273H mutant to suppress BMF expression that is dependent on constitutively active PI3K/AKT signaling. Collectively, these findings suggest that p53-R273H can specifically drive AKT signaling and suppress BMF expression, resulting in enhanced cell survivability and anoikis resistance. These findings open the possibility that blocking of PI3K/AKT will have therapeutic benefit in mutant p53-R273H expressing cancers.

The p53 protein is a tumor suppressor that functions as a sequence-specific transcription factor regulating the expression of various target genes involved in apoptosis, cell-cycle arrest, DNA repair, senescence, and inhibition of angiogenesis and metastasis.^1^ However, approximately 50% of all human cancers contain a mutation in the *TP53* gene, with the majority of these mutations occurring within the DNA-binding domain, causing an impaired binding of p53 to the DNA.^2, 3, 4, 5^ Unlike most tumor-suppressor genes, which are predominantly inactivated by deletions or truncating mutations during cancer progression, the *TP53* gene in human tumors is often found to undergo missense mutations that produce a full-length protein containing only a single amino acid substitution with a greatly prolonged half-life.^6, 7^

Most of the cancer-associated *TP53* mutations can be ascribed to two main classes: DNA contact and conformational mutants. The first group includes mutations in residues directly involved in DNA binding (e.g., R248Q and R273H). The second group comprises mutations that cause local (e.g., R249S and G245S) or global conformational distortions (e.g., R175H and R282W).^8, 9, 10^ The biological consequences of p53 mutations range from the mere loss-of-function to gain-of-function. Many *in vitro* studies have clearly demonstrated that some p53 mutants can acquire new functions, thereby contributing actively to the tumor initiation, progression and the increased resistance to conventional anticancer treatments.^3, 10, 11, 12, 13^ Indeed, mice knocked in with mutant p53-R270H or p53-R172H, corresponding to the human hotspot p53-R273H and p53-R175H mutants, respectively, developed highly metastatic tumors compared with p53-null mice, supporting the notion of gain-of-function properties acquired by mutant p53.^14, 15, 16, 17, 18, 19^

At the molecular level, several mechanisms have been suggested to account for mutant p53 gain-of-function including transcriptional activation of MYC, BAG1, MDR1, NFκB2, EGR1, GEF-H1, ID4 and MAD1;^20, 21, 22, 23, 24, 25, 26, 27, 28, 29^ transcriptional repression of ATF3, CD-95, ID2, hTERT and MST1;^30, 31, 32, 33^ unique interaction with specific DNA motives such as the nuclear matrix/scaffold attachment regions;^34^ epigenetic modification,^35^ regulation of miRNA^36, 37, 38^ and interactions with other proteins (e.g., p63, p73, NFY and BRD1).^39, 40, 41, 42^

Previous studies from our laboratories have demonstrated that a subset of tumor-derived p53 mutants mediate cell survival in breast cancer cells that expressed them.^43^ We found that silencing of mutant p53-R273H in MDA-MB-468 cells induced massive apoptosis.^43^ Importantly, the apoptotic effects following mutant p53 knockdown were independent of TAp63 and TAp73 function. Although considerable evidence is available documenting potential mechanisms through which p53 mutants deregulate cell growth, the mechanisms through which mutant p53 proteins enhance tumor cell survival remain relatively unexplored.

In the present study, therefore, we have investigated the effects of gain-of-function p53 mutants on deregulation of cell survival. We found that the p53-R273 contact mutant, but not the p53-R175 conformational mutant, promotes cancer cell survival and resistance to anoikis of cancer cells. Underlying these activities is the ability of p53-R273H mutant to suppress BMF expression in a way that is dependent on PI3K/AKT signaling pathway. Our results, thus, provided yet another mechanism as to how the mutant p53 proteins can contribute to diverse oncogenic and pro-metastatic signaling.

## Results

### Knockdown of endogenous p53-R273H contact mutant, but not R175H conformational mutant, induces mitochondria-dependent apoptosis

**Figure 1:**
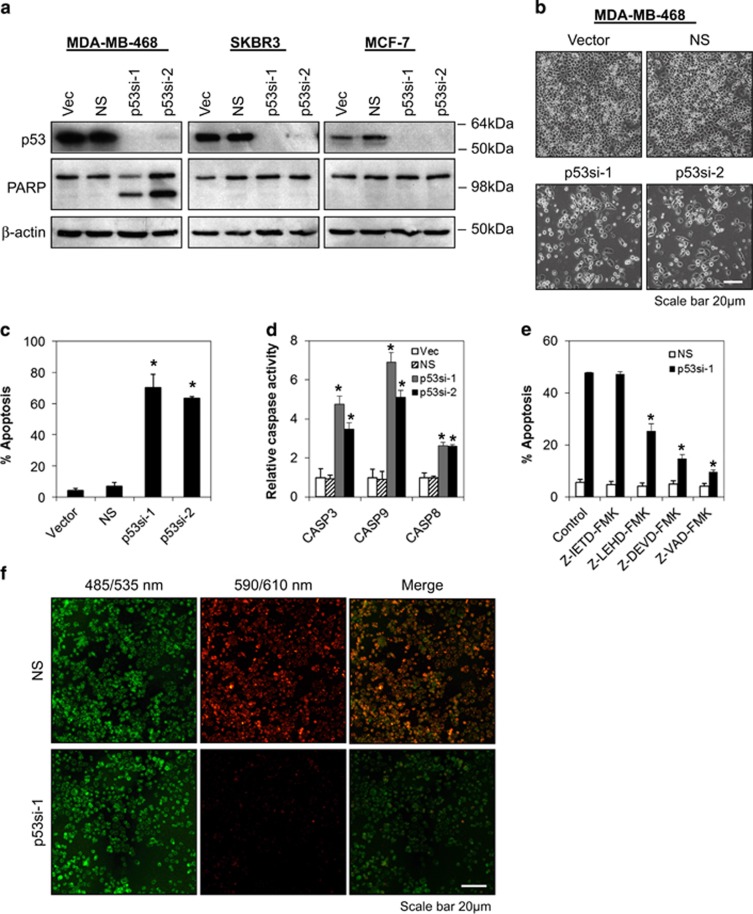
Knockdown of endogenous p53-R273H contact mutant, but not R175H conformational mutant, induces mitochondria-dependent apoptosis. (**a**–**c**) Mutant p53-R273H is required for the survival of human breast cancer cells. Cells were transduced with control vector (vec), non-targeting (NS) and two different lentiviral constructs that specifically target human p53 (p53si-1 and p53si-2). Lysates were prepared for immunoblotting at 72 h after transduction. *β*-actin serves as a loading control. Morphological changes were observed at 96 h after lentiviral transduction by light microscopy (original magnification × 100). Apoptotic cell death was determined using annexin V/7-AAD flow cytometry at 96 h after transduction. (**d**) Knockdown of mutant p53 induces caspase 8, 9 and 3 activation. Caspase activities were assessed by CaspaseGlo assay at 72 h following transduction. (**e**) Depletion of mutant p53 in MDA-MB-468 cells induces apoptosis which requires caspase 9 and 3, but not caspase 8, activities. Caspase-dependent cell death was evaluated by annexin V/7-AAD flow cytometry in the presence or absence of 20 *μ*M of caspase inhibitor following mutant p53-R273H knockdown. (**f**) Knockdown of mutant p53 in MDA-MB-468 cells induces mitochondrial membrane depolarization. Cells were stained with JC-1 at 72 h after p53 lentiviral shRNA transduction. Red color indicates the presence of JC-1 aggregates in intact mitochondria. Green color indicates JC-1 monomers in the cytoplasm. JC-1 stained cells were analyzed using epifluorescence microscopy (original magnification × 100). Bars represent mean±S.D. of three experiments. * indicates statistical significance (*P*<0.05) by Student's *t*-test

To determine the functional roles of p53 mutants in human breast cancer cells, endogenous p53 gene was silenced using lentiviral shRNA transduction. As shown in Figures 1a and c and Supplementary Figure 1, the silencing of endogenous p53-R273H contact mutant p53 by two independent p53-specific lentiviral shRNAs in MDA-MB-468 (breast), HT29 (colon) and A431 (epidermoid) cells induced massive apoptotic cell death as evidenced by PARP cleavage, cell blebbing and annexin V/7-AAD staining. In addition, caspase 3, caspase 9 and, to a lesser extent, caspase 8 activities were significantly upregulated following mutant p53-R273H knockdown in MDA-MB-468 cells (Figure 1d). Inhibition of caspase 9, caspase 3 and pan-caspase activities blocked the apoptosis induced by silencing of p53-R273H, whereas inhibition of caspase 8 activity did not rescue the cells from undergoing apoptosis, suggesting that the induction of apoptosis following p53-R273H depletion required caspase 9 and caspase 3, but not caspase 8 activity (Figure 1e). Consistently, depletion of endogenous p53-R273H in MDA-MB-468 cells also induced a significant amount of mitochondrial depolarization (Figure 1f). In contrast, no apoptotic effects were observed in MCF-7 cells that express wild-type p53, nor in SKBR3 cells that express p53-R175H conformational mutant (Figure 1a and Supplementary Figure 1c). These results suggest that endogenous p53-R273H contact mutant is mediating the survival of breast cancer cells by suppressing the mitochondrial apoptotic pathway.

### Depletion of mutant p53-R273H induces BCL2-modifying factor (BMF)

**Figure 2:**
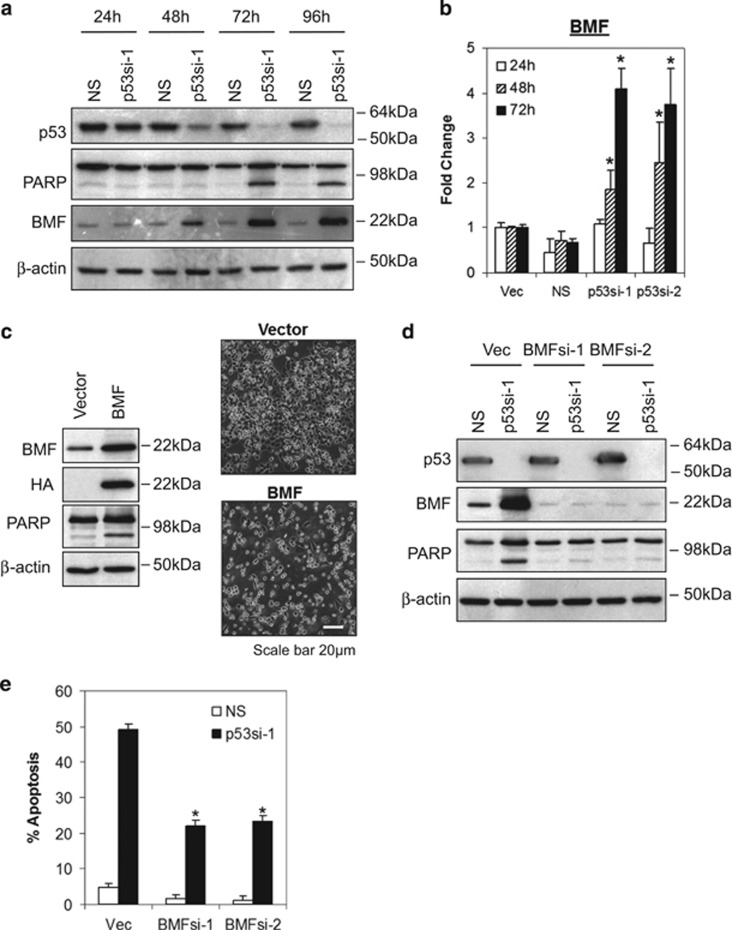
Depletion of mutant p53-R273H induces BMF. Knockdown of mutant p53 induces (**a**) BMF protein and (**b**) mRNA expression. MDA-MB-468 cells were transduced with p53 lentiviral shRNA. Protein and mRNA expression were accessed by immunoblot and quantitative RT-PCR, respectively. BMF mRNA expression was normalized against human GAPDH. (**c**) Ectopic expression of BMF is sufficient to induce apoptosis in MDA-MB-468 cells. Cells were transfected with 5 *μ*g of HA-tagged BMF using X-tremeGENE HP DNA transfection reagent as discussed in the Materials and Methods section. (**d**–**e**) Depletion of BMF rescues MDA-MB-468 cells from apoptosis induced by mutant p53 knockdown. Protein lysates and apoptosis were analyzed by (**d**) immunoblotting and (**e**) annexin V/7-AAD flow cytometry at 72 h after transduction. Bars represent mean±S.D. of three experiments. * indicates statistical significance (*P*<0.05) by Student's *t*-test

Because BCL2 family proteins have been shown to have a critical role in mitochondria-dependent apoptotic pathway, we hypothesize that mutant p53-R273H might mediate the survival of breast cancer cells through regulation of BCL-2 family proteins expression. To test this hypothesis, we investigated the expression of both pro- and anti-apoptotic BCL2 family proteins for their role in mitochondria-dependent apoptotic pathway. As shown in Figure 2a and Supplementary Figure 1a, one of the pro-apoptotic BCL2 family proteins, BMF, was upregulated at 48 h following depletion of endogenous p53-R273H, preceding the apoptotic event which occurred at 72 h (as indicated by PARP cleavage). In contrast, no significant difference in the expression of other pro-apoptotic (e.g., p-BAD, BAD, BAX, BID, BIM, PUMA and BOK) or anti-apoptotic (e.g., BCL-X_L_ and MCL-1) BCL-2 family members were observed (Supplementary Figure 2).

To further investigate whether upregulation of BMF occurred at the transcriptional level, real-time RT-PCR was performed to determine the level of BMF mRNA expression in MDA-MB-468 cells after p53-R273H knockdown. As shown in Figure 2b, knockdown of p53-R273H significantly induced BMF mRNA expression at 48 and 72 h, corroborated with the upregulation of the protein levels. In contrast, no such induction was observed in the p53-R175H-expressing SKBR3 cells (data not shown). Notably, the suppression of BMF expression by mutant p53-R273H in breast cancer cells is independent of direct promoter binding, as no endogenous p53-R273H was detected at the BMF promoter by chromatin immunoprecipitation assay (Supplementary Figure 3).

### BMF is required for the induction of apoptosis following depletion of endogenous p53-R273H

In order to test whether upregulation of BMF alone could induce apoptosis, ectopic expression of BMF was performed in MDA-MB-468 cells by transient transfection. As shown in Figure 2c, ectopic expression of BMF induces significant amount of apoptosis, suggesting that, indeed, overexpression of BMF could induce apoptosis in MDA-MB-468 cells.

We further investigated whether apoptosis induced by depletion of p53-R273H required endogenous BMF function. Our result showed that depletion of p53-R273H induced significant BMF upregulation in the vector control cells corroborated with significant apoptotic cell death (Figures 2d and e and Supplementary Figure 4); the levels of apoptosis were significantly reduced in BMF-depleted cells, suggesting that induction of apoptosis following p53-R273H depletion required BMF function.

### Induction of BMF sensitizes the release of BIM from BCL-X_L_ to promote mitochondrial apoptosis

**Figure 3:**
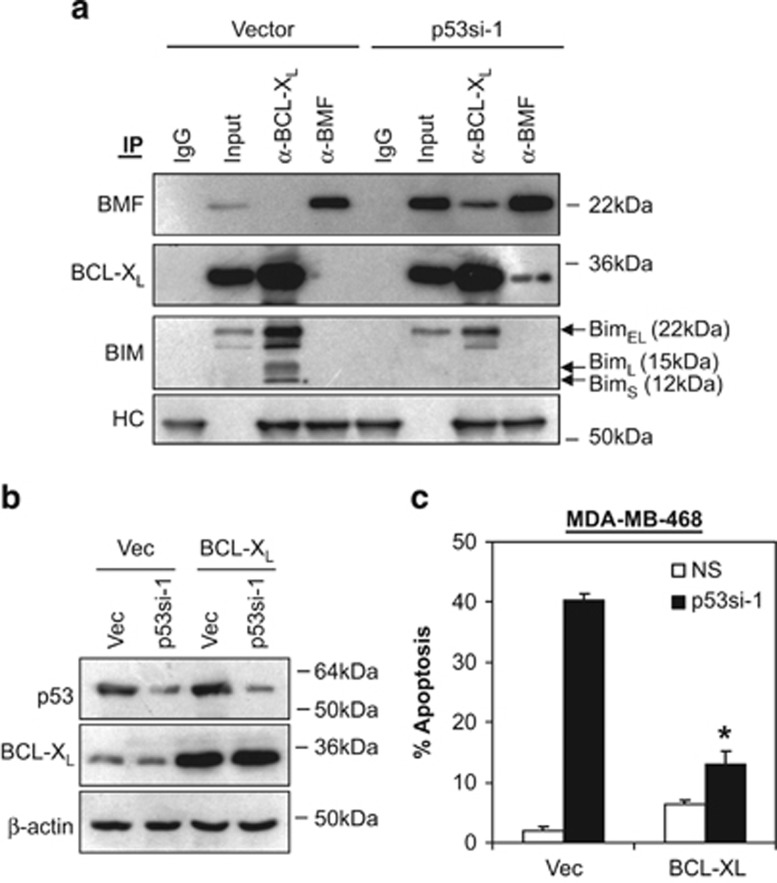
Induction of BMF sensitizes the release of BIM from BCL-X_L_. (**a**) MDA-MB-468 cells were transduced with non-targeting shRNA (NS) or p53si-1 lentiviral shRNA for 72 h and the interaction of BMF/BCL-X_L_ and BIM/BCL-X_L_ was detected by co-immunoprecipitation assay. Inputs for co-immunoprecipitation were also subjected to immunoblot analysis. IgG and heavy chain (HC) were used as a negative control and loading control, respectively. (**b**–**c**) Overexpression of BCL-X_L_ abrogated the apoptotic effects of mutant p53-R273H knockdown in MDA-MB-468 cells. Cells were co-transfected with BCL-X_L_ expression construct and p53si-1 shRNA. Protein lysates and apoptosis were analyzed by immunoblotting and annexin V/7-AAD flow cytometry at 72 h after co-transfection. Bars represent mean±S.D. of three experiments. *indicates statistical significance (*P*<0.05) by Student's *t*-test as compared with vector control cells

Various studies showed that certain damage signals, such as loss of cell attachment, will release BMF, allowing it to translocate, and bind to the pro-apoptotic BH3-only proteins, BIM, displacing pro-survival BCL2 or BCL-X_L_, to induce apoptosis.^44, 45, 46^ Here, we investigated whether the upregulation of BMF following depletion of endogenous p53-R273H in MDA-MB-468 cells might induce BCL-X_L_/BMF hetero-dimerization and disrupt BCL-X_L_/BIM interaction. Indeed, our results demonstrated that BCL-X_L_ bound to BIM but not BMF under normal conditions (Figure 3a). However, upon induction of BMF following p53-R273H knockdown, a significant amount of BMF was found to bind BCL-X_L_, coinciding with the dissociation of BIM from BCL-X_L_ (Figure 3a). Consistently, overexpression of BCL-X_L_ completely abrogated the apoptotic effects of p53-R273H depletion (Figures 3b and c). Of note, no BCL-2 was detected in MDA-MB-468 cells. Together, these results suggest that silencing of endogenous mutant p53-R273H in MDA-MB-468 cells induced BMF expression and the formation of BMF/BCL-X_L_ complexes. This in turn leads to the displacement of BIM from BCL-X_L_ to induce apoptosis.

### Mutant p53-R273H suppresses cellular anoikis

**Figure 4:**
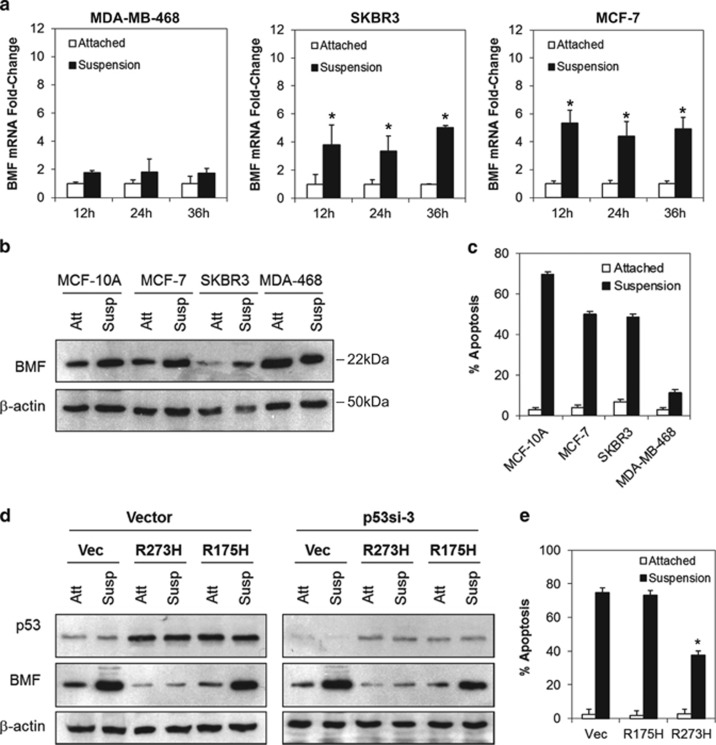
Mutant p53-R273H suppresses cellular anoikis. (**a**–**b**) Endogenous p53-R273H suppresses BMF induction in breast cancer cells cultured in suspension. Total RNA samples and protein lysates derived from cells cultured as attached monolayers or in suspension were subjected to real-time RT-PCR analysis and immunoblotting, respectively. (**c**) p53-R273H expressing MDA-MB-468 cells were inherently resistant to anoikis. All cells were cultured as in **a.** Apoptosis/anoikis was determined using annexin V/7-AAD flow cytometry. (**d**) Ectopic expression of p53-R273H, but not p53-R175H, suppresses BMF protein and mRNA induction (Supplementary Figure 5c) in MCF-10A cells cultured in suspension. MCF-10A p53si-3 cells were transfected with vector, p53-R175H- or p53-R273H-expressing plasmid. Protein lysates derived from cells cultured as attached monolayers (att) or in suspension (susp) were subjected to immunoblotting. (**e**) Ectopic expression of p53-R273H confers anoikis resistance in MCF-10A cells. MCF-10A p53si-3 cells were cultured and transfected as in **d**. Apoptosis was analyzed by annexin V/7-AAD flow cytometry at 48 h after culture. Bar represents mean±S.D. of three independent experiments. * indicates statistical significancet (*P*<0.05) by Student's *t*-test

Because our results showed that endogenous p53-R273H mediates the survival of breast cancer cells through suppression of BMF and that BMF has been reported to mediate cellular anoikis,^44, 47^ we hypothesized that p53-R273H could also exert its oncogenic function through the suppression of cellular anoikis in cancer cells. To test this hypothesis, we examined the sensitivity of a number of cancer cell lines harboring different p53 mutations to anoikis. As shown in Table 1, more than 50% apoptotic cell death was observed in cancer cell lines expressing wild-type p53 or various mutants (R280T, R175H, R179R, L194F and M246I) in suspension culture. In contrast, cancer cells expressing p53-R273H (MDA-MB-468, HT29 and A431) exhibit significant resistance to anoikis. Similarly, significant BMF mRNA and protein induction was also observed in the anoikis-sensitive MCF-7 and MCF-10A (wild-type p53), and SKBR3 (p53-R175H) cells, whereas no significant BMF induction was observed in the anoikis-resistant MDA-MB-468, HT29 and A431 cells which harbor p53-R273H mutation (Figures 4a and c and Supplementary Figure 5a and b). These results suggest that p53-R273H mutant might inhibit cellular anoikis through suppression of BMF induction.

To directly validate whether the p53-R273H contact mutant was indeed capable of suppressing BMF expression and anoikis, and not because of the inherent anoikis resistance of the MDA-MB-468, HT29 or A431 cells, we performed a gene reconstitution experiment in the non-transformed MCF-10A cells. A pool of MCF-10A cells stably transduced with a lentiviral p53 shRNA targeting the endogenous 3′-UTR of the wild-type p53 (p53si-3) was generated followed by ectopic transient expression of the p53-R175H or p53-R273H mutants. The levels of anoikis were then analyzed by culturing the transfected cells on poly-HEMA-treated tissue culture plates followed by annexin V/7-AAD flow cytometry analysis.

As shown in Figure 4d, more than 80% of the endogenous p53 expression was downregulated in cells stably expressing p53si-3 shRNA in MCF-10A cells. As expected, detachment of cells induced significant upregulation of BMF mRNA and protein levels in the vector control cells and the p53-R175H expressing MCF-10A (Figure 4d and Supplementary Figure 5c). In contrast, no such upregulation of BMF was observed in cells transfected with p53-R273H mutant, suggesting that p53-R273H, but not p53-R175H, suppresses BMF expression in MCF-10A cells growing in suspension. Consistently, p53-R273H-expressing MCF-10A cells were significantly more resistant to anoikis as compared with vector or p53-R175H-expressing cells (*P*<0.05, Student's *t*-test) (Figure 4e). Similar results were also observed in p53-null H1299 lung cancer cells (Supplementary Figures 5d and e), suggesting that the p53-R273H contact mutant, but not the p53-R175H conformational mutant, confers anoikis resistance in human cancer cells.

### Mutant p53-R273H regulates PI3K/AKT signaling pathway

In order to identify the mechanism by which mutant p53-R273H mediates tumor cell survival and anoikis resistance, microarray gene profiling was conducted following endogenous p53-R273H depletion in MDA-MB-468 cells. A total of 58 genes were upregulated (>1.5 fold-change), while 117 genes were downregulated at 72 h following p53-R273H knockdown (Supplementary Figure 6). Of note, the microarray results show that BMF expression was upregulated while p53 mRNA was downregulated following knockdown of p53-R273H in MDA-MB-468 cells, independently validating our previous results which demonstrated that depletion of p53-R273H induces BMF mRNA and protein expression.

To validate the microarray results, we performed independent real-time RT-PCR on a number of candidate genes. Indeed, the expression of LZTFL1, POU2F3 and SOSTDC1 was upregulated in a time-dependent manner following p53-R273H knockdown in MDA-MB-468 cells, while TMBIM1, ICAM1 and LIFR were significantly downregulated, consistent with the microarray data (Supplementary Figure 6).

Having ascertained the p53-R273H gene signature, we sought to identify the direct molecular target of p53-R273H using the Connectivity Map resource.^48, 49^ The Connectivity Map consists of pattern-matching software that compares an input gene signature to a database of 7000 expression profiles representing 1309 small molecules (termed perturbagens). A connectivity score from +1 to −1 is then assigned based on the degree of similarity or dissimilarity between the two signatures.^48, 49^ Thus, a drug with a high connectivity score and low *P* value has a gene signature very similar to the query signature and might be hypothesized to act on a pathway in parallel with the drug that generated the query signature.

The Connectivity Map analysis shows that three of the top five perturbagens predicted to share common pathways activated by p53-R273H depletion in MDA-MB-468 cells were either direct or indirect inhibitors of PI3K signaling pathway (Table 2). These include wortmannin and LY-294002, which are PI3K inhibitors,^50^ and sirolimus, an mTOR inhibitor.^51^ In addition, we also identified vorinostat and trichostatin A, both are histone deacetylase inhibitors, as one of the hits following p53-R273H knockdown. However, unlike the PI3K inhibitors, the gene signature that was enriched following p53-R273H knockdown was inversely correlated with the gene signatures induced by histone deacetylase inhibitors. These findings led us to hypothesize that p53-R273H might exert its gain-of-function effects through activation of PI3K/AKT signaling pathway and/or inhibition of histone deacetylase activities.

### Depletion of p53-R273H dephosphorylates AKT

To directly test this hypothesis, immunoblotting was performed to evaluate the expression of phosphorylated AKT following p53-R273H knockdown in MDA-MB-468 cells.

**Figure 5:**
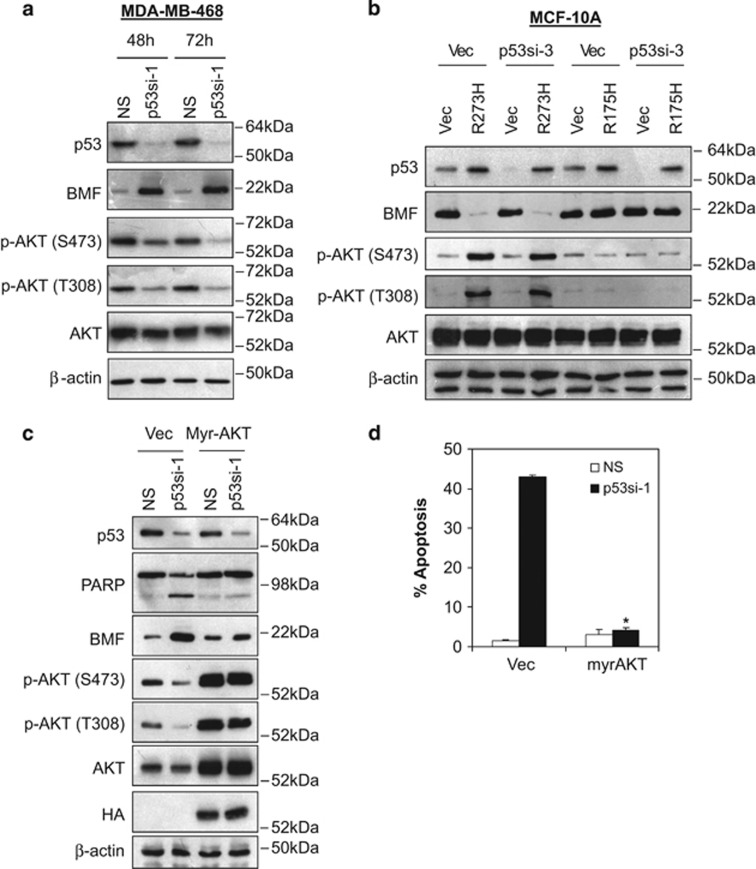
Mutant p53-R273H regulates PI3K/AKT signaling pathway. (**a**) Knockdown of p53-R273H in MDA-MB-468 cells reduces AKT phorphorylation. MDA-MB-468 cells were transduced with non-targeting (NS) or p53si-1 lentiviral shRNA and lysates were isolated for immunoblotting analysis at 48 and 72 h after transduction. (**b**) Ectopic expression of mutant p53-R273H, but not p53-R175H, induces AKT phosphorylation. MCF-10A cells were transduced with lentiviral vector or p53si-3 shRNAs targeting the 3′-UTR of the endogenous wild-type p53 followed by brief drug selection (1 *μ*M of puromycin). Pool of p53si-3 stably expressing cells 3 were transfected with vector, p53-R175H or p53-R273H followed by immunoblotting at 72 h after transfection. (**c**) Ectopic expression of myr-AKT suppresses BMF expression following p53-R273H knockdown in MDA-MB-468 cells. (**d**) Ectopic expression of myr-AKT abrogated the apoptotic effects induced by p53-R273H depletion in MDA-MB-468 cells. Cells were co-transfected with myr-AKT and p53si-1 for 72 h. Apoptosis was determined using annexin V/7-AAD flow cytometry. Bar represents mean±S.D. of three independent experiments

As shown in Figure 5a and Supplementary Figure 7a, induction of BMF following depletion of endogenous p53-R273H corroborated with the reduction of AKT phosphorylation at serine 473 and threonine 308. Similarly, ectopic expression of p53-R273H in MCF-10A p53si-3 or H1299 cells induced significant AKT phosphorylation and suppression of BMF, whereas no such effects were observed in cells transfected with p53-R175H, nor in cells transfected with vector control (Figure 5b and Supplementary Figure 7b ).

We further investigated the dependence of p53-R273H-mediated BMF suppression and cell survival on AKT activity. Indeed, the expression of a constitutively active myristoylated AKT completely abrogated the induction of BMF and dramatically reduced apoptosis following p53-R273H depletion in MDA-MB-468, HT29 and A431 cells (Figures 5c and d and Supplementary Figures 7c and d). Together, these results demonstrate that p53-R273H mutant is capable of regulating PI3K and AKT signaling.

### Mutant p53-R273H regulates specifically AKT and BMF expression in a wide range of cancer cells

**Figure 6:**
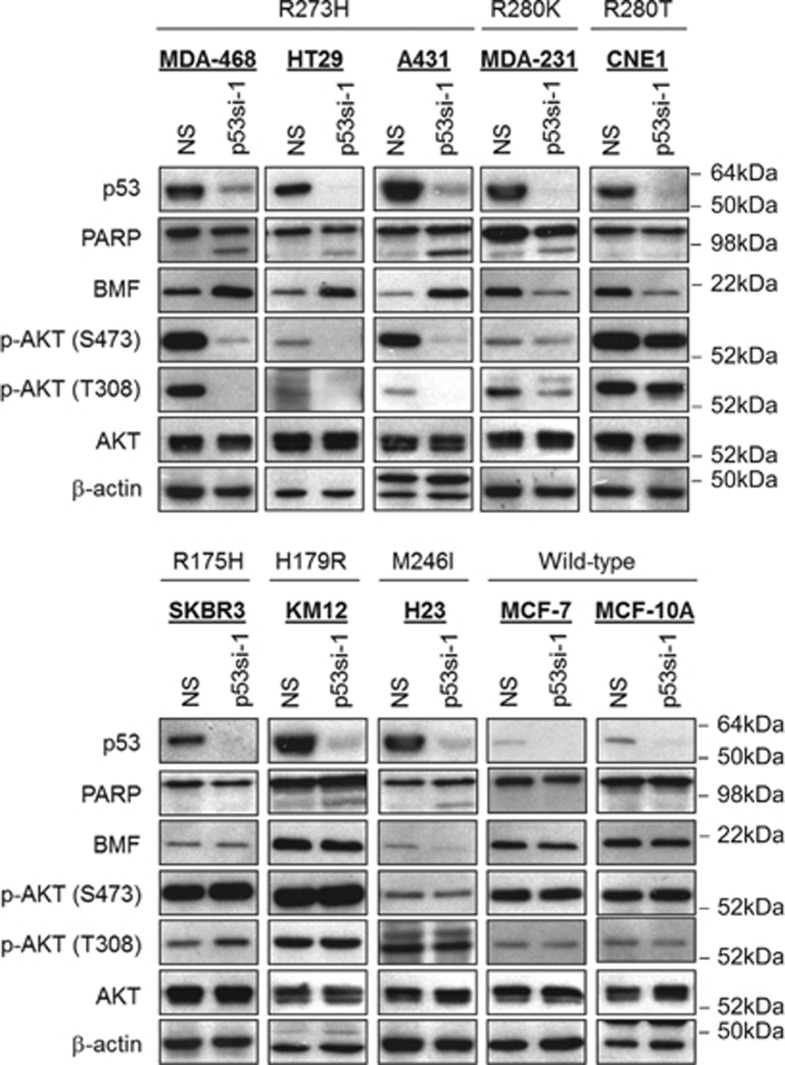
Mutant p53-R273H regulates AKT and BMF expression in a wide range of cancer cells. Cells were transduced with p53si-1 lentiviral shRNA. Protein lysates were isolated for immunoblotting at 72 h after transduction. Note the dephosphorylation of AKT corroborated with BMF induction only in cells bearing p53-R273H.

To determine whether the pro-survival effect of mutant p53-R273H is present in other tumor types, we performed p53 gene knockdown in a panel of human cancer cell lines harboring various p53 mutations. As shown in Figure 6, depletion of endogenous p53-R273H in MDA-MB-468, HT29 and A431 induced significant amount of apoptosis and BMF expression, consistent with our previous observations.

Intriguingly, depletion of endogenous p53-R280K in MDA-MB-231 and p53-R280T in CNE-1 cells also reduced AKT phosphorylation, but instead of upregulation, BMF expression was found to be significantly downregulated and no apoptotic cell death was observed. In stark contrast, depletion of p53-M246I in NCI-H23 lung cancer cells led to BMF downregulation with unchanged phospho-AKT levels. The levels of phosho-AKT and BMF remained unaffected in SKBR3 (p53-R175H), KM12 (p53-H179R), MCF-7 (wild-type p53) or MCF-10A (wild-type p53) following endogenous p53 knockdown. These results suggest that mutant p53-R273H, but not other mutants, specifically regulate tumor cell survival through AKT-dependent suppression of AKT.

### p53 mutant correlates with AKT phosphorylation in primary breast tumors

Because our results predict that tumors with mutant p53 would show more active PI3K/AKT signaling, we stained tissue microarrays of human breast cancer samples for p53 and compared this with the levels of AKT phosphorylation. We used high p53 staining as an indication of the presence of potential gain-of-function p53 mutations (Supplementary Figure 6). Consistent with our model, a significant positive correlation (*P*<0.001, Chi-square test) was detected between elevated p53 staining and high levels of AKT phosphorylation (Supplementary Figure 8). Collectively, these results suggest that p53 mutants specifically drive cancer cell survival and anoikis resistance through alteration of AKT signaling.

## Discussion

This study describes a gain-of-function of mutant p53-R273H in promoting both cell survival and anoikis resistance of cancer cells. Underlying these activities is the ability of mutant p53-R273H to suppress BMF expression in a way that is dependent on PI3K/AKT signaling pathway. Depletion of endogenous p53-R273H hot-spot contact mutant, but not the p53-R175H conformational mutant, downregulated AKT phosphorylation, induced BMF expression, sensitized BIM dissociation from BCL-X_L_ and induced significant mitochondria-dependent apoptosis in a wide range of cancer cells. Importantly, the knockdown of BMF or ectopic expression of a myristoylated AKT significantly rescued the apoptotic effects of p53-R273H depletion, suggesting that BMF induction is required for the apoptotic cell death following p53-R273H knockdown. Similarly, cancer cells harboring endogenous p53-R273H mutant were also found to be inherently resistant to anoikis and lack of BMF induction following culture in suspension. As predicted by this model, overexpression of p53-R273H, but not p53-R175H, in the p53 wild-type MCF-10A cells rendered the cells resistant to anoikis and suppresses BMF induction. These results suggest that mutant p53-R273H specifically drives cancer cell survival through suppression of BMF, which might contribute to increased anoikis resistance during metastasis. Because induction of BMF was also abolished in myristoylated AKT-expressing cells following p53-R273H knockdown, it seems likely that the induction of BMF is downstream of AKT inhibition.

A further demonstration of the clinical relevance of the p53 mutants and PI3K/AKT axis was provided by IHC staining of primary human breast cancers showing a positive correlation between high expression of p53 (an indicator of the presence of gain-of-function mutant p53) and strong phospho-AKT staining.

It should be cautioned, though, that the effect of mutant p53 on PI3K/AKT signaling is not limited to only p53-R273H mutants. The results also do not necessarily imply that all tumors having high phospho-AKT levels would possess p53-R273H mutation and have the same degree of resistance to apoptosis and anoikis. Indeed, depletion of endogenous p53-R280K in MDA-MB-231 and p53-R280T in CNE-1 cells confers similar downregulation of phospho-AKT, but BMF expression was found to be significantly downregulated (instead of upregulated as observed in p53-R273H-expressing cells) and no apoptotic cell death was observed. It seems likely that other p53 mutants might cooperate via different mechanisms, independent of BMF, to convey different oncogenic signals to promote aggressiveness in human cancers. This warrants further investigation.

How, exactly, p53 mutants contribute to PI3K/AKT activation remains an open question. Because p53 gain-of-function mutants can drive tumorigenesis through activation of growth factor receptors (e.g., TGF-*β* receptor,^52^ EGFR^53, 54, 55^ and MET^56, 57^) or promote integrin/Rab-coupling protein-driven recycling.^53^ These signaling pathways have been implicated in the activation of PI3K/AKT signaling. Hence, it is tempting to speculate that the constitutive activation of PI3K/AKT by p53-R273H may be partly modulated by one or a combination of these mechanisms. Indeed, the three cell lines (MDA-MB-468, HT29 and A431) that were responsive to AKT de-phosphorylation, BMF induction and apoptotic cell death following p53-R273H depletion, express moderate to high levels of EGFR.^58^ Further work is indeed necessary to understand whether the role of mutant p53-R273H in the regulation of PI3K/AKT signaling and BMF expression influences metastatic development in human cancers. Research addressing this aspect will also need to ascertain the relative contribution of each functional property of mutant p53-R273H, that is, control of apoptosis, cell migration and cell invasion, in tumorigenesis.

Finally, several studies have also revealed a role for TAp63, a p53 family protein and transcription factor, which interacts with mutant but not wild-type p53.^59, 60^ By inhibiting TAp63, mutant p53 can regulate a pro-invasive transcription program that includes regulation of the expression of Dicer, DEPDC1, Cyclin G2 and Sharp1.^52, 61^ However, our findings suggest that mutant p53-R273H can regulate cancer cells survival and anoikis resistance independent of the TAp63 (and TAp73) functions.^43^ This is consistent with a previous study which shows that the loss of TAp63 is less potent in inducing metastasis as compared with mutant p53 expression in a mouse model of pancreatic ductal adenocarcinoma, suggesting that mutant p53 does more than inhibiting TAp63.^62^

In conclusion, we demonstrated that p53-R273H contact mutant suppresses BMF expression in a way that is dependent on PI3K/AKT signaling pathway to promote cancer cell survival and anoikis resistance. Our results, thus, provide evidence for another mechanism that tumors may use to promote metastasis, especially in tumors where mutant p53 is highly expressed. These findings also open the possibility that targeting PI3K/AKT signaling pathway will have therapeutic benefit in mutant p53-R273H expressing cancers.

## Materials and Methods

### Cell culture and constructs

Human breast carcinoma cell lines (HCC38, MCF-7, MDA-MB-231, MDA-MB-468 and SKBR3), colon carcinoma cell lines (HT-29 and KM12), lung carcinoma cell lines (NCI-H1299 and NCI-H23), epidermoid carcinoma (A431) and nasopharyngeal carcinoma (CNE1) cell lines were maintained in RPMI 1640 containing 10% fetal bovine serum, 100 IU/ml penicillin, and 100 *μ*g/ml streptomycin (Sigma-Aldrich, St Louis, MO, USA). MCF-10A cells were grown in DMEM-F12 (Sigma-Aldrich) supplemented with 5% horse serum, 20 ng/ml EGF, 0.5 g/ml hydrocortisone, 100 ng/ml cholera toxin, 10 *μ*g/ml insulin, 100 IU/ml penicillin and 100 *μ*g/ml streptomycin. All cells were maintained at 37 °C in an environment containing 5% CO_2_. Expression constructs were obtained from Addgene (Cambridge, MA, USA) and were transfected using X-tremeGENE HP DNA transfection reagent (Roche, Indianapolis, IN, USA) according to the manufacturer's instruction (Supplementary Table 1).

### Lentiviral production and transduction

Lentiviral shRNA constructs were purchased from Sigma-Aldrich. High-titer lentiviral stocks were generated by co-transfection with packaging plasmids psPAX2 (Addgene; plasmid 12260) and envelope plasmids pMD2.G (Addgene; plasmid 12259) into HEK-293T cells as described previously.^43, 63, 64, 65, 66^ The shRNA target sequences for p53 and BMF are shown in Supplementary Table 2.

### Immunoblot analysis

Protein lysates from cells were extracted in ice-cold lysis buffer (0.75% NP-40, 1 mM DTT, phosphatase inhibitors and protease inhibitors in PBS). Total protein (50 *μ*g) was subjected to SDS-PAGE followed by immunoblotting. Details of the source of antibodies used for immunoblotting are provided in the Supplementary Table 3.

### Apoptosis and anoikis assay

Quantitation of apoptosis by Annexin V-PE/7-AAD staining was performed as described previously.^43, 63^ All analysis was performed on a FACSCalibur flow cytometer using CellQuest Pro software (version 5.1.1; BD Biosciences, San Jose, CA, USA). For anoikis assay, cells were plated on tissue culture plates pretreated with 20 mg/ml of polyHEMA (Sigma, St. Louis, MO, USA) and analyzed for apoptosis by Annexin V-PE assay.

### Caspase activation and inhibition

Caspase 3, 8 and 9 activities in cells were measured at 72 h after lentiviral shRNAs transduction using a CaspaseGlo kit (Promega, Madison, WI, USA) according to the manufacturer's protocol. Caspase 3, 8, 9 or pan-caspase inhibitor (Z-DEVD-FMK, Z-IETD-FMK, Z-LEHD-FMK and Z-VAD-FMK, respectively; Promega) was used to inhibit caspase activity. The optimum concentration of all inhibitors was determined to be 20 *μ*M. Inhibitors were added to cells 24 h after lentiviral shRNA transduction. Apoptosis was measured by Annexin V-PE assay at 72 h.

### Mitochondrial membrane depolarization assay

Cells were incubated with 5 μg/ml of JC-1 (Merck, Darmstadt, Germany) in dark condition for 30 min at room temperature followed by visualization with fluorescence microscopy or flow cytometry analysis.

### Quantitative RT-PCR

Total RNA from cells was extracted using Qiagen RNA isolation kit (Qiagen, Valencia, CA, USA) and first-strand cDNA was synthesized using High Capacity RNA-to-cDNA Master Mix (Applied Biosystems, Carlsbad, CA, USA) according to the manufacturers' protocol. Gene expression levels were measured by real-time RT-PCR using the FastStart Universal SYBR Green Master reagent (Roche) and a Bio-Rad iQ5 real-time PCR detector system (Bio-Rad, Richmond, CA, USA). Data analysis was performed using Bio-Rad iQ5 Optical System Software V1.0. The specific forward and reverse primer sequences are shown in Supplementary Table 4. The conditions for all real-time RT-PCR reactions were as follows: 3 min at 94 °C followed by 40 s at 94 °C, 40 s at 60 °C and 25 s at 72 °C for 40 cycles. The expression data were normalized against GAPDH or B2M as housekeeping gene.

### Microarray and connectivity map analysis

Microarray experiments were performed in duplicate in MDA-MB-468 cells transduced with p53 lentiviral shRNA or with non-specific shRNA control for 48 h. Isolated RNA was submitted to a Affymetrix certified service laboratory (Origen Labs, Singapore) for quality control and processing. All hybridization was performed on a Affymetrix Human Gene 1.0ST Array. Microarray data analyses were performed using the Affymetrix Expression Console V1.1 (Santa Clara, CA, USA), Gene Set Enrichment Analysis (Broad Institute of Harvard and MIT, MA, USA)^67^ and the Connectivity Map.^48^ All microarray expression data have been deposited in the National Center for Biotechnology Information's Gene Expression Omnibus (GEO series accession number: GSE65967).

## Figures and Tables

**Table 1:** Cancer cells harboring mutant p53-R273H are inherently resistant to anoikis in suspension cell culture

**Tumor type**	**Cell line**	**p53 status**	**Apoptotic cells, %±S.D.**	
			**Attached**	**Suspension**
Breast	MDA-MB-468	R273H	0.12±0.05	14.43±2.43
Colon	HT29	R273H	0.49±0.23	13.28±2.56
Epidermoid	A431	R273H	0.56±0.23	37.05±2.89
Breast	HCC38	R273L	1.55±0.40	59.70±0.92
Breast	SKBR3	R175H	1.65±0.12	52.24±2.94
Colon	KM12	H179R	0.72±0.08	64.75±3.78
Nasopharynx	CNE1	R280H	0.79±0.21	75.87±3.58
Breast	T47D	L194F	0.11±0.04	88.82±0.25
Lung	H23	M246I	0.91±0.13	80.98±1.62
Breast	MCF-7	WT	1.80±1.02	67.70±2.18
Breast	MCF-10A	WT	0.68±0.43	72.28±0.87

**Table 2:** Top 25 pharmaceutical perturbagens identified through the Connectivity Map that induce a genes signature following p53-R273H depletion

**Rank**	**Pharmaceutical pertubagen**	***P*****-value**	**Mean connectivity score**^a^	**Description**
1	Vorinostat	<1.0E-05	−0.450	Histone deacetylase inhibitors
2	Wortmannin	<1.0E-05	0.261	PI3Ks inhibitor
3	Trichostatin A	<1.0E-05	−0.413	Histone deacetylase inhibitors
4	Sirolimus	<1.0E-05	0.297	mTOR inhibitor
5	LY-294002	<1.0E-05	0.352	PI3Ks inhibitor
6	Prestwick-664	1.1E-03	0.440	−
7	AG-013608	1.7E-03	0.423	−
8	Amikacin	1.9E-03	0.559	Aminoglycoside antibiotic
9	Mometasone	2.0E-03	−0.471	Glucocorticosteroid
10	H-7	2.1E-03	−0.458	−
11	Phenoxybenzamine	2.4E-03	0.510	Alpha antagonist
12	Liothyronine	3.0E-03	0.553	Thyroid hormone
13	Betazole	3.2E-03	−0.345	Histamine H2 receptor agonist
14	Demecarium bromide	3.6E-03	−0.412	Acetylcholinesterase inhibitor
15	Piroxicam	4.3E-03	0.430	NSAID
16	Nalbuphine	5.0E-03	−0.277	Analgesic
17	0179445-0000	5.1E-03	0.255	−
18	Mianserin	6.2E-03	−0.426	Tetracyclic antidepressant
19	Naphazoline	8.1E-03	0.389	Vasoconstrictor
20	Felodipine	8.1E-03	−0.249	Calcium channel blocker
21	Guaifenesin	8.7E-03	0.449	Expectorant drug
22	Ikarugamycin	1.0E-02	−0.447	Antibiotic
23	Tomatidine	1.1E-02	0.326	Steroidal alkaloid
24	Caffeic acid	1.2E-02	0.580	−
25	Deferoxamine	1.2E-02	0.355	Chelating agent

a Mean connectivity score among all treatment instances

## Abbreviations

7-AAD: 7-aminoactinomycin D
AKT: v-akt murine thymoma viral oncogene homolog 1
ATF3: activating transcription factor 3
B2M: beta-2-microglobulin
BAD: BCL2-associated agonist of cell death
BAG1: BCL2-associated athanogene
BAX: BCL2-associated X protein
BCL-2: B-cell CLL/lymphoma 2
BCL-X_L_: BCL2-like 1 (BCL2L1)
BID: BH3 interacting domain death agonist
BIM: BCL2-like 11 (apoptosis facilitator) (BCL2L11)
BMF: Bcl2-modifying factor
BOK: BCL2-related ovarian killer
BRD1: bromodomain containing 1
CD-95: Fas cell surface death receptor (also known as FAS)
DEPDC1: DEP domain containing 1
Dicer: dicer 1, ribonuclease type III
DTT: Dithiothreitol
EGF: epidermal growth factor
EGFR: epidermal growth factor receptor
EGR1: early growth response 1
GAPDH: glyceraldehyde-3-phosphate dehydrogenase
GEF-H1: Rho/Rac guanine nucleotide exchange factor (GEF) 2 (also known as ARHGEF2)
