# Supplementary material for: Mutant p53-R273H mediates cancer cell survival and anoikis resistance through AKT-dependent suppression of BCL2-modifying factor (BMF)
Source: Cell Death Dis. 2015 Jul 16;6(7):e1826–. doi: 10.1038/cddis.2015.191 (PMC4650736; doi:10.1038/cddis.2015.191)
Supplement: Supplementary Figure 1 [file cddis2015191x1.ppt]

## Slide 1
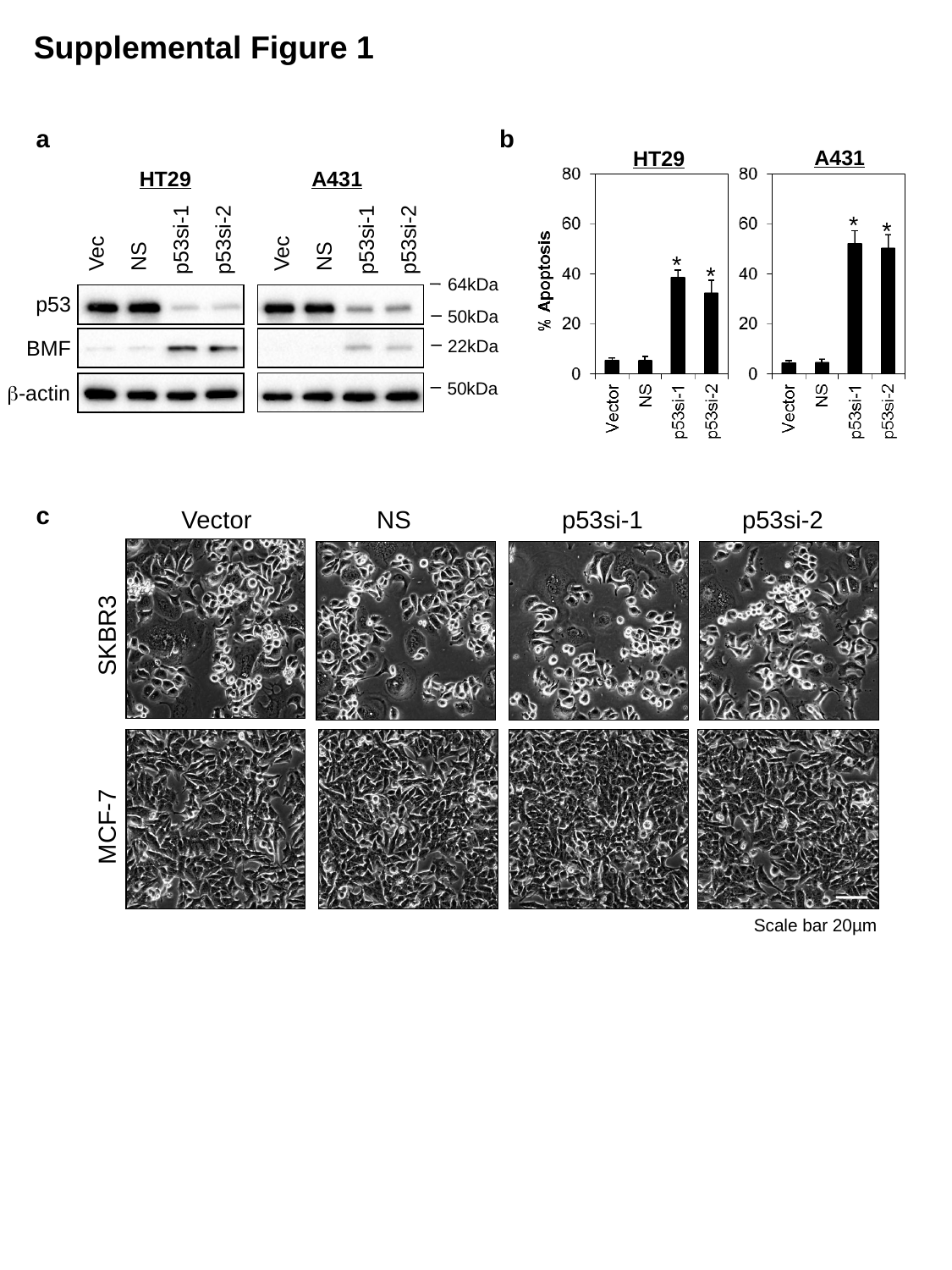

Supplemental Figure 1
b
a
A431
HT29
HT29
A431
*
*
p53si-1
p53si-2
p53si-1
p53si-2
Vec
Vec
NS
NS
*
*
 64kDa
p53
 50kDa
BMF
22kDa
 50kDa
β-actin
c
Vector
NS
p53si-1
p53si-2
SKBR3
MCF-7
Scale bar 20µm
