# Supplementary material for: Mutant p53-R273H mediates cancer cell survival and anoikis resistance through AKT-dependent suppression of BCL2-modifying factor (BMF)
Source: Cell Death Dis. 2015 Jul 16;6(7):e1826–. doi: 10.1038/cddis.2015.191 (PMC4650736; doi:10.1038/cddis.2015.191)
Supplement: Supplementary Figure 2 [file cddis2015191x2.ppt]

## Slide 1
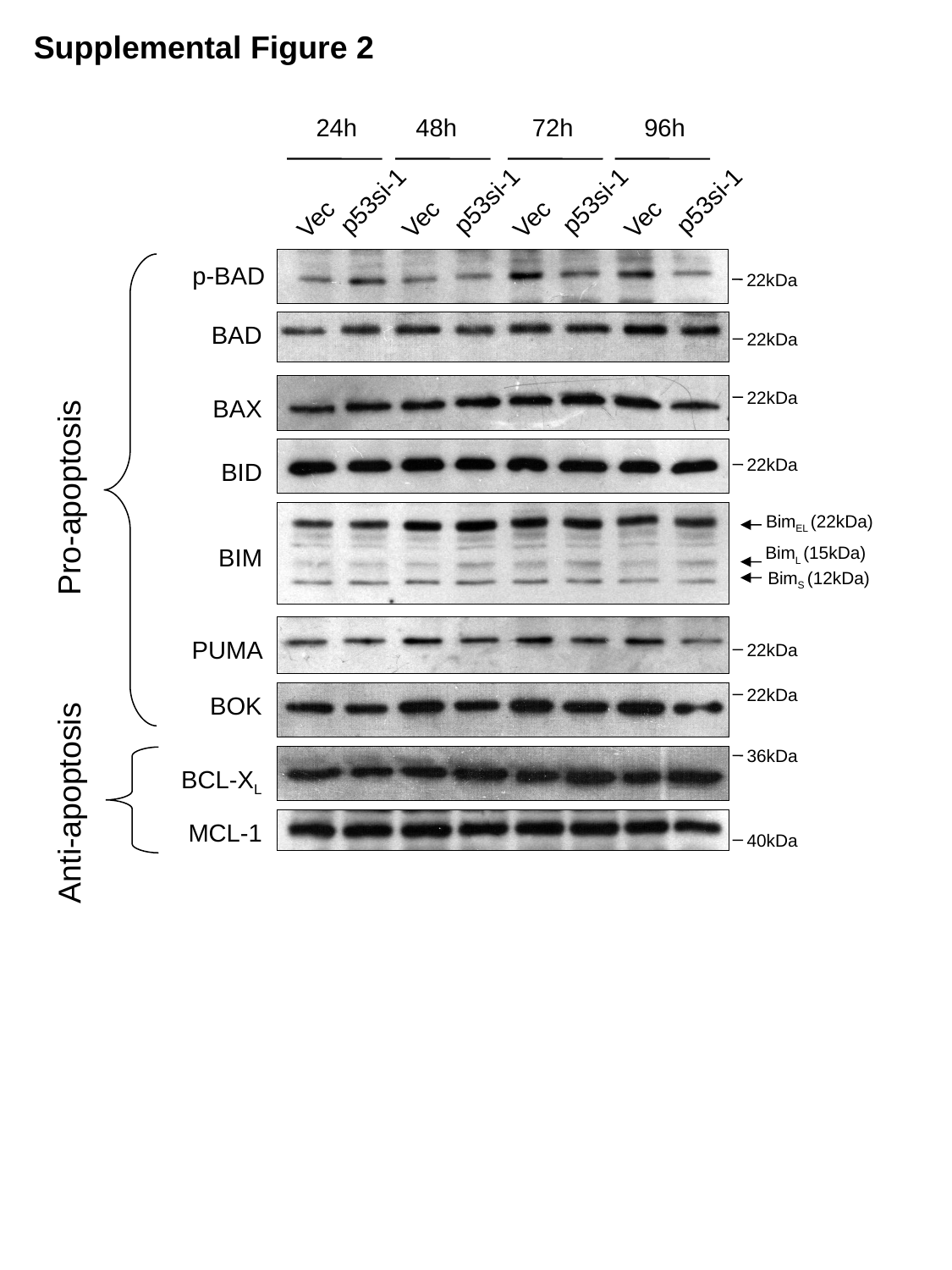

Supplemental Figure 2
24h
48h
72h
96h
p53si-1
p53si-1
p53si-1
p53si-1
Vec
Vec
Vec
Vec
p-BAD
22kDa
BAD
22kDa
22kDa
BAX
22kDa
BID
Pro-apoptosis
BimEL (22kDa)
BIM
BimL (15kDa)
BimS (12kDa)
PUMA
22kDa
22kDa
BOK
36kDa
BCL-XL
Anti-apoptosis
MCL-1
40kDa
