# Supplementary figures and images for: Mutant p53-R273H mediates cancer cell survival and anoikis resistance through AKT-dependent suppression of BCL2-modifying factor (BMF)
Source: Cell Death Dis. 2015 Jul 16;6(7):e1826–. doi: 10.1038/cddis.2015.191 (PMC4650736; doi:10.1038/cddis.2015.191)

## Slide 1
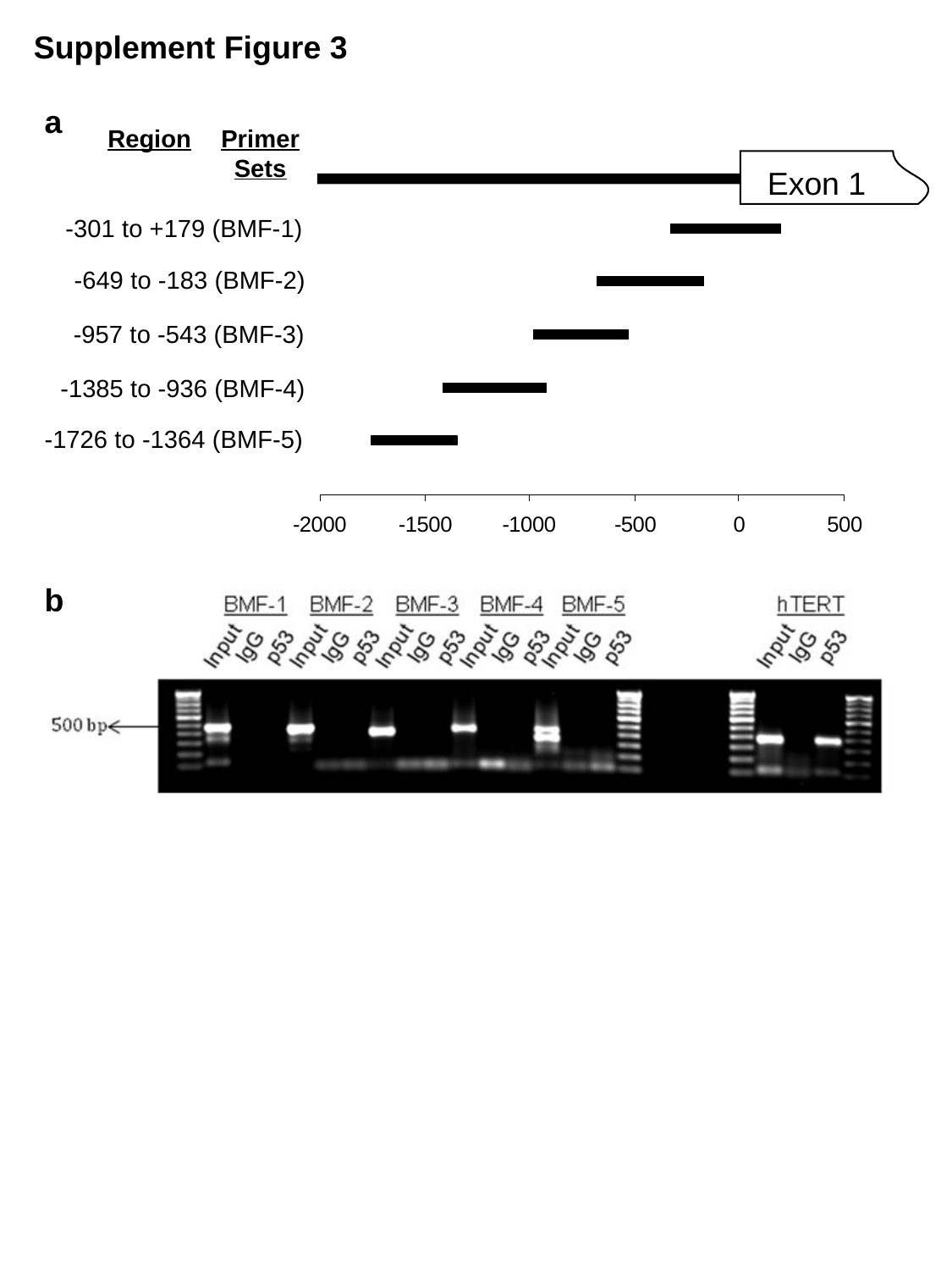

Supplement Figure 3
Exon 1
a
Region
Primer
Sets
-301 to +179 (BMF-1)
-649 to -183 (BMF-2)
-957 to -543 (BMF-3)
-1385 to -936 (BMF-4)
-1726 to -1364 (BMF-5)
b

Supplement: Supplementary Figure 3 [file cddis2015191x3.ppt]

## Slide 1
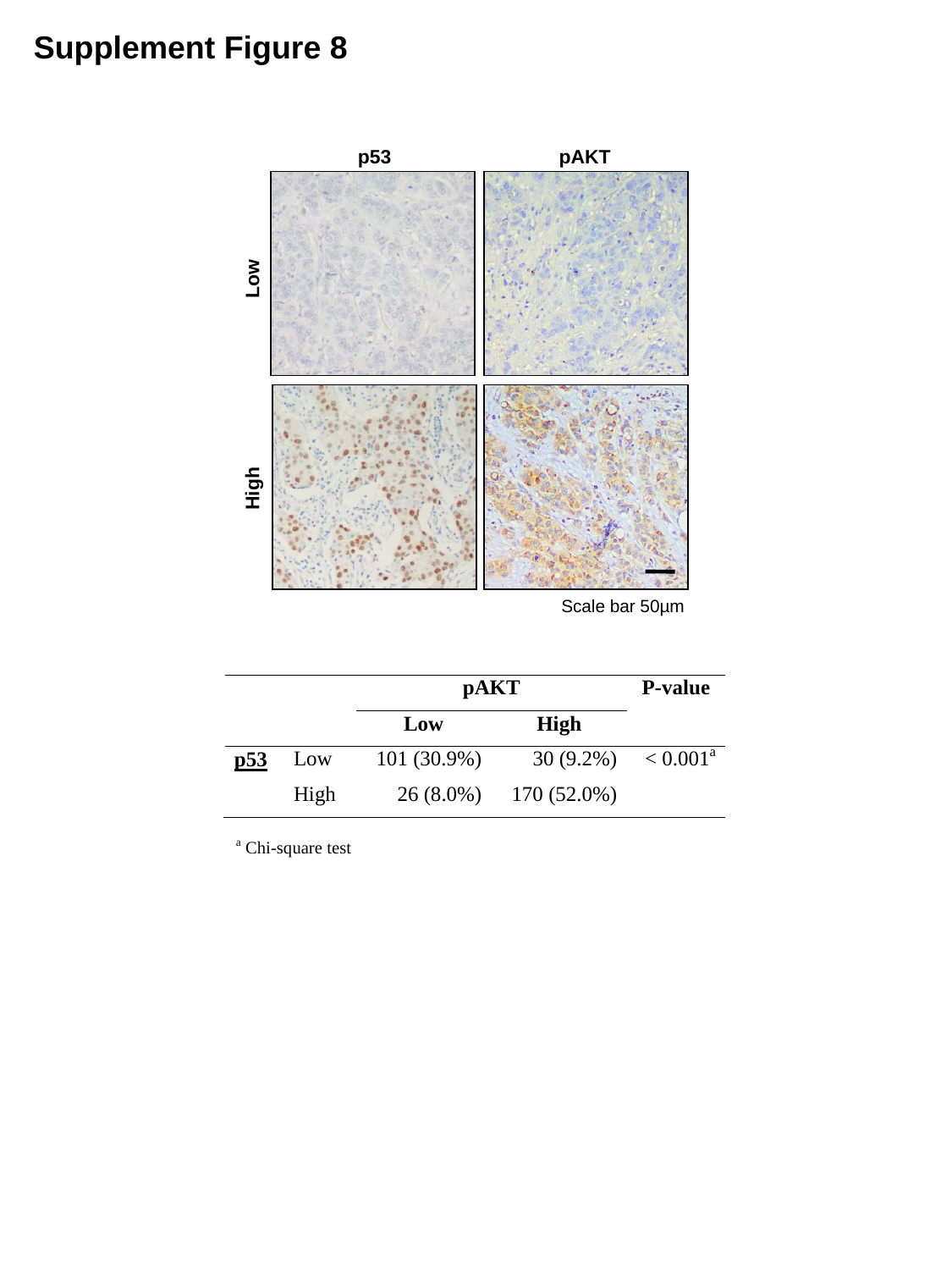

Supplement Figure 8
p53
pAKT
Low
High
Scale bar 50µm

Supplement: Supplementary Figure 8 [file cddis2015191x8.ppt]
