# Supplementary material for: Mutant p53-R273H mediates cancer cell survival and anoikis resistance through AKT-dependent suppression of BCL2-modifying factor (BMF)
Source: Cell Death Dis. 2015 Jul 16;6(7):e1826–. doi: 10.1038/cddis.2015.191 (PMC4650736; doi:10.1038/cddis.2015.191)
Supplement: Supplementary Figure 4 [file cddis2015191x4.ppt]

## Slide 1
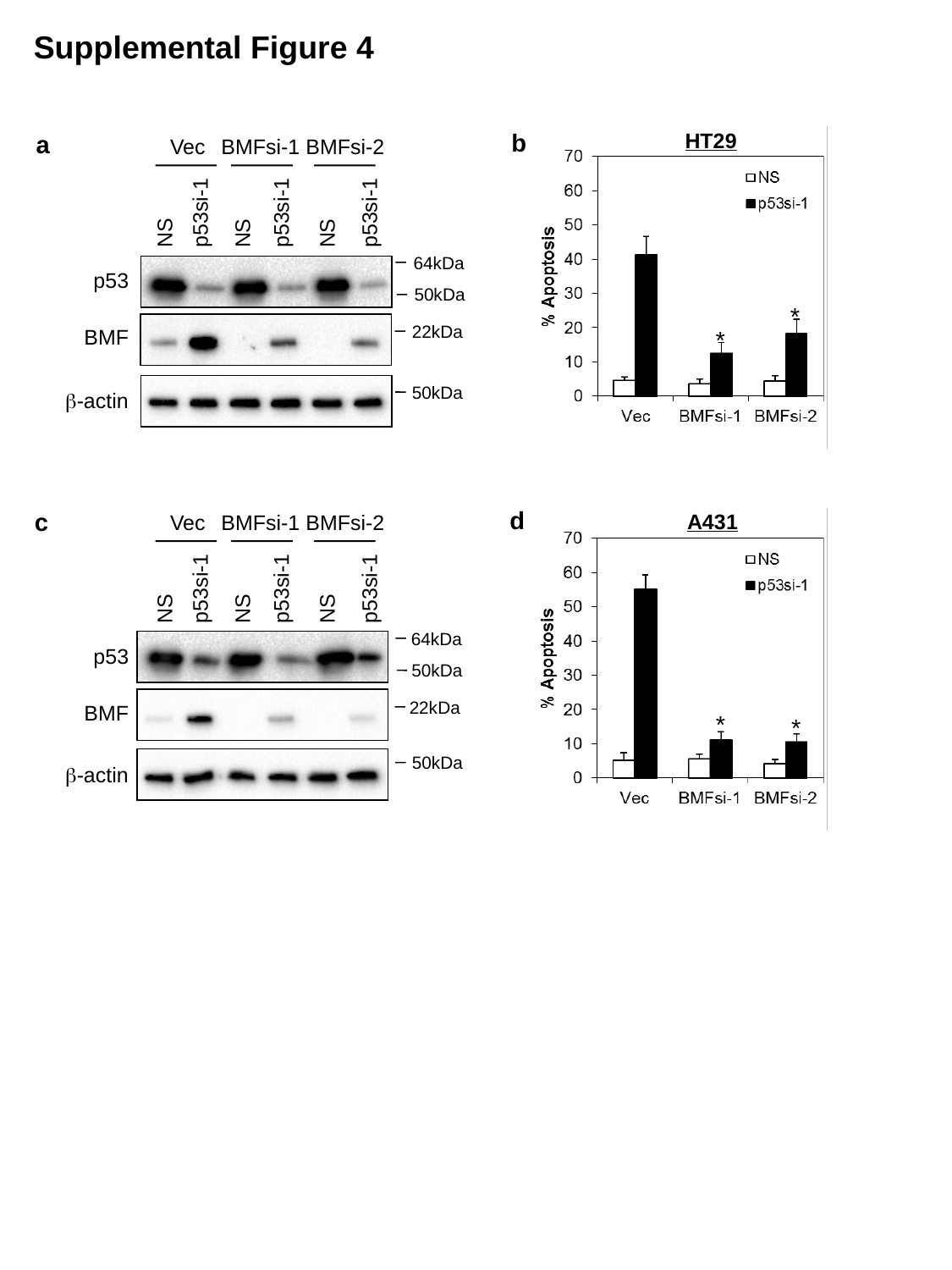

Supplemental Figure 4
b
HT29
a
Vec
BMFsi-1
BMFsi-2
p53si-1
p53si-1
p53si-1
NS
NS
NS
64kDa
p53
50kDa
*
22kDa
BMF
*
50kDa
β-actin
d
c
A431
Vec
BMFsi-1
BMFsi-2
p53si-1
p53si-1
p53si-1
NS
NS
NS
64kDa
p53
50kDa
22kDa
BMF
*
*
50kDa
β-actin
