# Supplementary material for: Mutant p53-R273H mediates cancer cell survival and anoikis resistance through AKT-dependent suppression of BCL2-modifying factor (BMF)
Source: Cell Death Dis. 2015 Jul 16;6(7):e1826–. doi: 10.1038/cddis.2015.191 (PMC4650736; doi:10.1038/cddis.2015.191)
Supplement: Supplementary Figure 5 [file cddis2015191x5.ppt]

## Slide 1
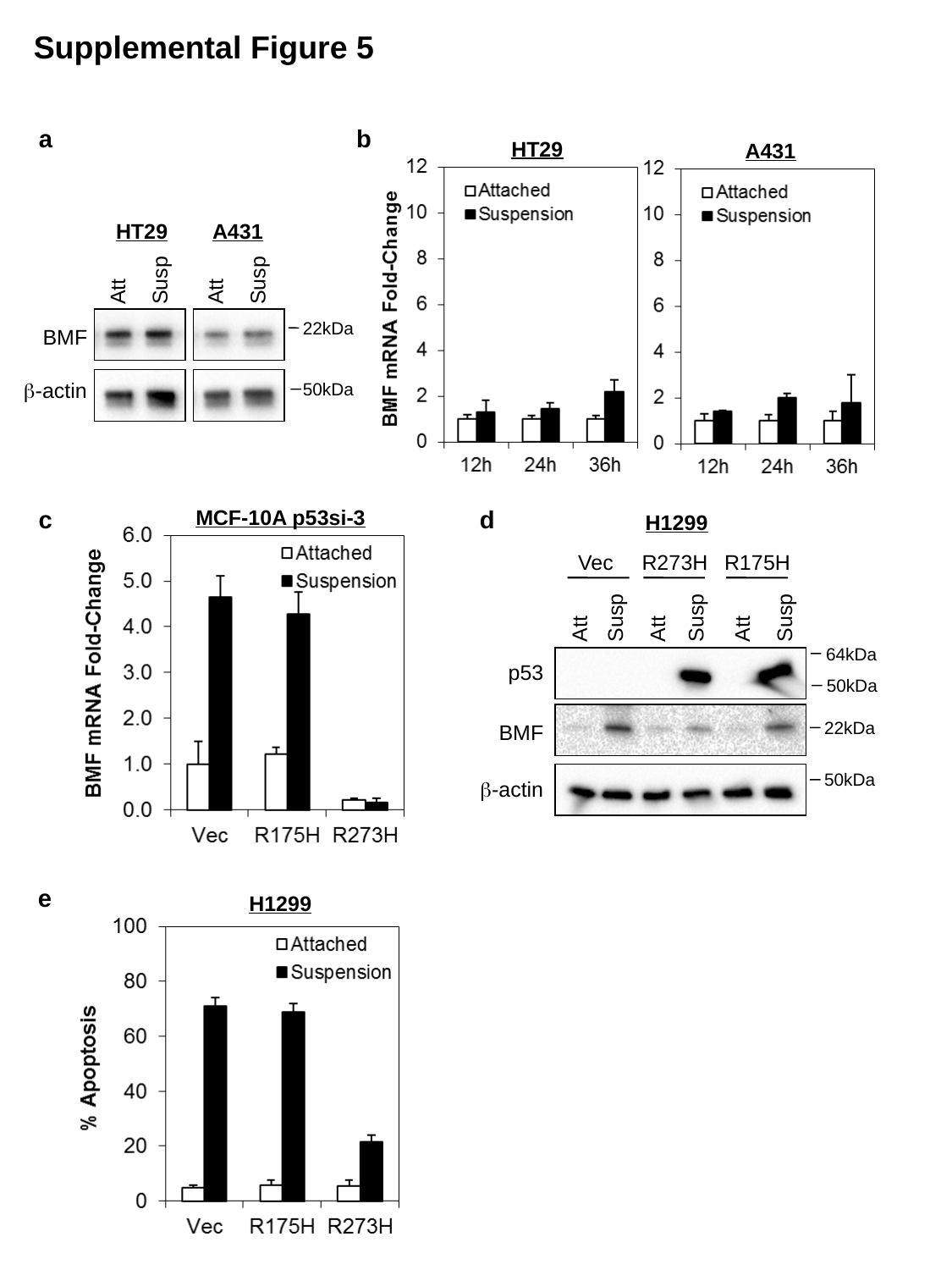

Supplemental Figure 5
b
a
HT29
A431
HT29
A431
Susp
Susp
Att
Att
22kDa
BMF
β-actin
50kDa
c
MCF-10A p53si-3
d
H1299
Vec
R273H
R175H
Susp
Susp
Susp
Att
Att
Att
64kDa
p53
50kDa
22kDa
BMF
50kDa
β-actin
e
H1299
