# Supplementary material for: Mutant p53-R273H mediates cancer cell survival and anoikis resistance through AKT-dependent suppression of BCL2-modifying factor (BMF)
Source: Cell Death Dis. 2015 Jul 16;6(7):e1826–. doi: 10.1038/cddis.2015.191 (PMC4650736; doi:10.1038/cddis.2015.191)
Supplement: Supplementary Figure 6 [file cddis2015191x6.ppt]

## Slide 1
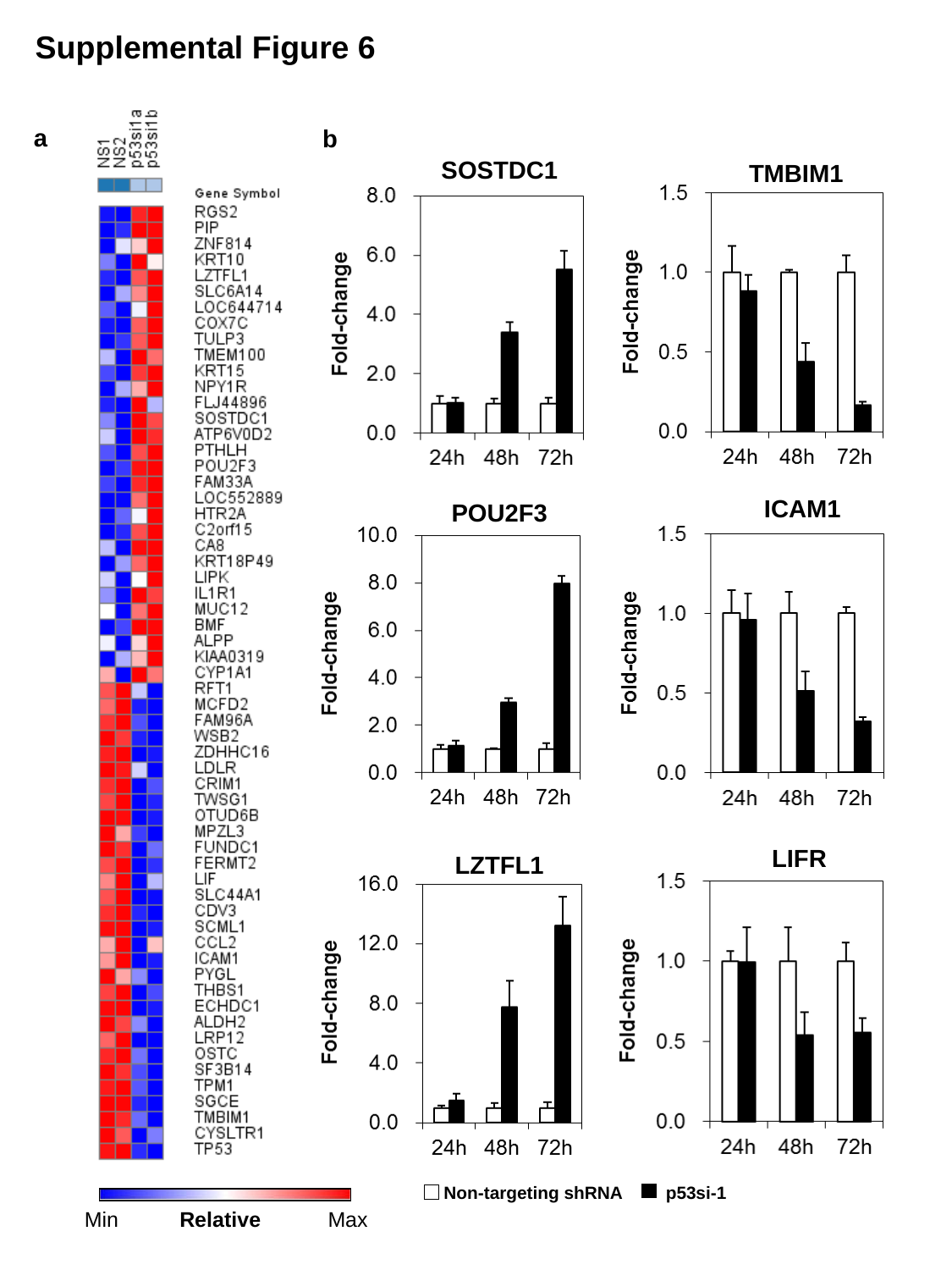

Supplemental Figure 6
a
b
SOSTDC1
TMBIM1
ICAM1
POU2F3
LIFR
LZTFL1
Non-targeting shRNA
p53si-1
Min
Relative
Max
