# Supplementary material for: Mutant p53-R273H mediates cancer cell survival and anoikis resistance through AKT-dependent suppression of BCL2-modifying factor (BMF)
Source: Cell Death Dis. 2015 Jul 16;6(7):e1826–. doi: 10.1038/cddis.2015.191 (PMC4650736; doi:10.1038/cddis.2015.191)
Supplement: Supplementary Figure 7 [file cddis2015191x7.ppt]

## Slide 1
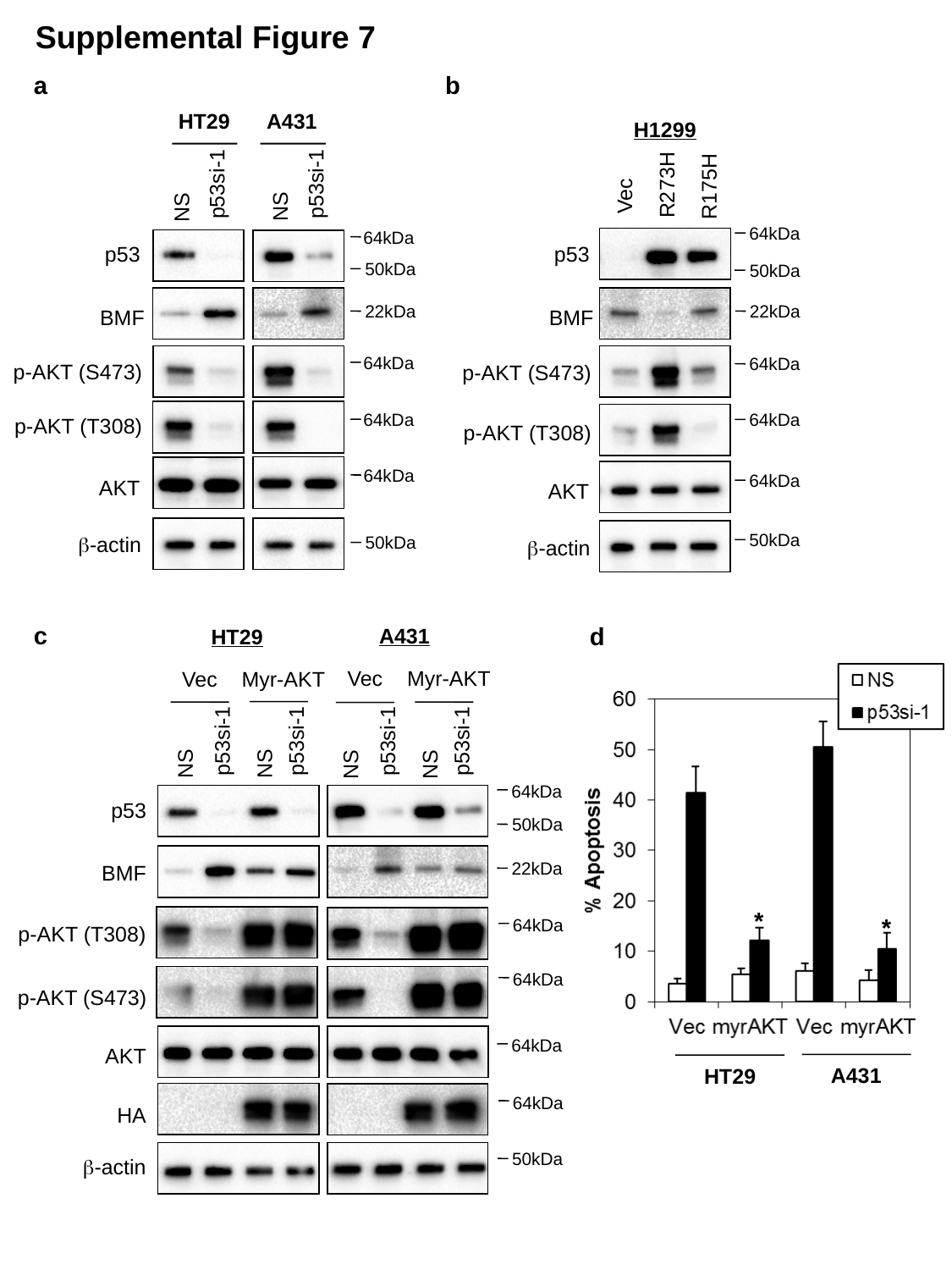

Supplemental Figure 7
a
b
HT29
A431
H1299
p53si-1
p53si-1
R273H
R175H
Vec
NS
NS
64kDa
64kDa
p53
p53
50kDa
50kDa
22kDa
22kDa
BMF
BMF
64kDa
64kDa
p-AKT (S473)
p-AKT (S473)
64kDa
64kDa
p-AKT (T308)
p-AKT (T308)
64kDa
64kDa
AKT
AKT
50kDa
β-actin
50kDa
β-actin
c
d
A431
HT29
Vec
Myr-AKT
Vec
Myr-AKT
p53si-1
p53si-1
p53si-1
p53si-1
NS
NS
NS
NS
64kDa
p53
50kDa
22kDa
BMF
*
*
64kDa
p-AKT (T308)
64kDa
p-AKT (S473)
64kDa
AKT
A431
HT29
64kDa
HA
50kDa
β-actin
