# Supplementary material for: Mutant p53-R273H mediates cancer cell survival and anoikis resistance through AKT-dependent suppression of BCL2-modifying factor (BMF)
Source: Cell Death Dis. 2015 Jul 16;6(7):e1826–. doi: 10.1038/cddis.2015.191 (PMC4650736; doi:10.1038/cddis.2015.191)
Supplement: Supplementary Figure Legends [file cddis2015191x9.docx]

**SUPPLEMENTAL FIGURE LEGENDS**

**Supplemental Figure 1: Knock-down of endogenous mutant p53-R273H in HT29 and A431 cells induces BMF expression and apoptosis. (a-b)** HT29 colorectal cancer and A431 epidermoid cancer cells were transduced with control vector (vec), non-targeting (NS) and 2 different lentiviral constructs that specifically target human p53 (p53si-1 and p53si-2). Lysates were prepared for immunoblotting at 72 h after transduction. Apoptotic cell death was determined using annexin V/7-AAD flowcytometry at 96 h after transduction. Bar represents mean ± s.d. of 3 independent experiments. * indicates statistical significancet (p<0.05) by Student’s t-test. **(c)** Morphological changes associate with knock-down of p53 in SKBR3 and MCF-7. Cells were transduced with vector and non-specific lentiviral shRNA as control, and p53-specific lentiviral shRNAs (p53si-1 and p53si-2) overnight. Morphological changes were observed at 96 h after lentiviral transduction by light microscopy (original magnification 100X).

**Supplemental Figure 2: Knock-down mutant p53-R273H has no effect on BAD, BAX, BID, BIM, PUMA, BOK, BCL-X_L_ or MCL-1 protein expression.** MDA-MB-468 cells were transduced with non-specific shRNA or p53si-1 lentiviral shRNA and their protein lysates isolated for immunoblotting analysis at 24, 48, 72 and 96 h after transduction as reported in Figure 2a. Endogenous BCL-2 and BAK were not detectable in MDA-MB-468 cells.

**Supplemental Figure 3: Endogenous p53-R273H mutant does not bind to BMF promoter in MDA-MB-468. (a)** Schematic representation of the BMF primer pairs spanning 1.5 kb up stream of the BMF transcriptional start site. **(b)** ChIP analysis showed that p53 R273H mutant does not bind to BMF promoter. IgG served as negative control and hTERT served as positive control. Note the binding of the mutant p53-R273H on the hTERT promoter, consistent with previous studies [^49^](#_ENREF_49)^,^ [^50^](#_ENREF_50), while no such binding was observed in the BMF promoter using the same samples.

**Supplemental Figure 4: Depletion of BMF rescues HT29 and A431cells from apoptosis induced by mutant p53-R273H knock-down. (a-b)** HT29 and **(c-d)** A431 cells were transduced with p53 lentiviral shRNA. Protein lysates and apoptosis were analyzed by immunoblotting and annexin V/7-AAD flow cytometry at 72 h after transduction. Bars represent mean ± s.d. of 3 experiments. * indicates statistical significance (P < 0.05) by Student’s t-test.

**Supplemental Figure 5: Mutant p53-R273H suppresses cellular anoikis. (a-b)** Endogenous p53-R273H suppresses BMF induction in HT29 and A431 cells cultured in suspension. **(c-e)** Ectopic expression of p53-R273H in MCF-10A p53-si3 and p53-null H1299 cells suppresses BMF mRNA expression and anoikis. Protein lysates derived from cells cultured as attached monolayers (att) or in suspension (susp) were subjected to immunoblotting. Apoptosis was analyzed by annexin V/7-AAD flow cytometry at 48 h after culture. Bar represents mean ± s.d. of 3 independent experiments. * indicates statistical significancet (p<0.05) by Student’s t-test.

**Supplemental Figure 6: Differential gene expression regulated by mutant p53-R273H in MDA-MB-468 cells.** **(a)** Heatmaps of the top 30 differentially regulated genes (fold change > 1.5) following p53-R273H knock-down in MDA-MB-468 cells NS1 and NS2, cells transduced with non-targeting shRNA; p53si1a and p53si1b, cells transduced with p53si-1 specific lentiviral shRNA. Both experiments were conducted independently. **(b)** Validation of microarray data by real-time RT-PCR. MDA-MB-468 cells were transduced with p53 lentiviral shRNA and the mRNA isolated similar to the procedure used for microarray analysis at the designated time points. mRNA was then converted to cDNA and measured by real-time RT-PCR. The left panel represents genes that are up-regulated as determined by microarray. The right panel represents genes that are down-regulated as determined by microarray. Bars represent the mean ± s.d. of at least three independent experiments.

**Supplemental Figure 7: Mutant p53-R273H regulates PI3K/AKT signaling pathway. (a)** Knock-down p53-R273H in HT29 and A431 cells reduces AKT phosphorylation. Cells were transduced with non-targeting (NS) or p53si-1 lentiviral shRNA and lysates were isolated for immunoblotting analysis at 72 h after transduction. **(b)** Ectopic expression of mutant p53-R273H, but not p53-R175H, in H1299 cells induces AKT phosphorylation. **(c-d)** Ectopic expression of myr-AKT suppresses BMF expression and apoptosis following p53-R273H knock-down in HT29 and A431 cells. Apoptosis was determined using annexin V/7-AAD flow cytometry. Bar represents mean ± s.d. of 3 independent experiments.

**Supplemental Figure 8: p53 mutant correlates with AKT phosphorylation in primary breast tumors.** Immunostaining of primary breast cancer sections with antibodies against p53 and phospho-AKT. Note the presence of nuclear staining for p53 and cytoplasmic staining for phospho-AKT.
