# Supplementary material for: Mutant p53-R273H mediates cancer cell survival and anoikis resistance through AKT-dependent suppression of BCL2-modifying factor (BMF)
Source: Cell Death Dis. 2015 Jul 16;6(7):e1826–. doi: 10.1038/cddis.2015.191 (PMC4650736; doi:10.1038/cddis.2015.191)
Supplement: Supplementary Tables [file cddis2015191x10.docx]

**SUPPLEMENTAL TABLES**

**Supplemental Table 1: Sources of expression plamids.**

| **Expression construct** | **Sources** |
| --- | --- |
| pCMV bam | Addgene plasmid 16440 |
| pCMV p53-R273H | Addgene plasmid 16439 |
| pCMV p53-R175H | Addgene plasmid 16436 |
| pCMV p53 wt | Addgene plasmid 16434 |
| pBabe-neo | Addgene plasmid 1767 |
| pBabe-BMF | Addgene plasmid 17239 |
| pcDNA3 | Addgene plasmid 20011 |
| pcDNA3 myr-Akt | Addgene plasmid 9008 |
| pMIG | Addgene plasmid 9044 |
| pMIG BCL-X_L_ | Addgene plasmid 8790 |

**Supplemental Table 2: shRNA target sequences.**

| **shRNA** | **Target sequences** |
| --- | --- |
| p53si-1 | 5’-CACCATCCACTACAACTACAT-3’ |
| p53si-2 | 5’-CGGCGCACAGAGGAAGAGAAT-3’ |
| p53si-3 | 5’-GAGGGATGTTTGGGAGATGTA-3’ |
| BMFsi-1 | 5’-GCTTTGAATGGAGAAGAGAAC-3’ |
| BMFsi-2 | 5’-CCAGAGGAACTCAGTTAAGAA-3’ |

**Supplemental Table 3: Primary antibodies for immunoblotting.**

| **Target** | **Clone** | **Manufacturer** | **Dilution** |
| --- | --- | --- | --- |
| p53 | DO-1 | Santa Cruz Biotechnology | 1:1000 |
| BMF | Polyclonal | Abcam | 1:1000 |
| PARP | 46D11 | Cell Signaling Technology | 1:1000 |
| HA | C29F4 | Cell Signaling Technology | 1:1000 |
| AKT | C67E7 | Cell Signaling Technology | 1:1000 |
| p-AKT (Ser 473) | D9E | Cell Signaling Technology | 1:1000 |
| p-AKT (Thr 308) | C31E5E | Cell Signaling Technology | 1:1000 |
| BCL-2 | 50E3 | Cell Signaling Technology | 1:1000 |
| BCL-X_L_ | 54H6 | Cell Signaling Technology | 1:1000 |
| MCL-1 | D35A5 | Cell Signaling Technology | 1:1000 |
| BAD | D24A9 | Cell Signaling Technology | 1:1000 |
| p-BAD (Ser 112) | 40A9 | Cell Signaling Technology | 1:1000 |
| BAX | D2E11 | Cell Signaling Technology | 1:1000 |
| BIK | Polyclonal | Cell Signaling Technology | 1:1000 |
| BIM | C34C5 | Cell Signaling Technology | 1:1000 |
| BID | Polyclonal | Cell Signaling Technology | 1:1000 |
| BAK | D2D3 | Cell Signaling Technology | 1:1000 |
| PUMA | Polyclonal | Cell Signaling Technology | 1:1000 |
| β-actin | C-2 | Santa Cruz Biotechnology | 1:250 |

**Supplemental Table 4: Forward and reverse primer sequences for**

**quantitative RT-PCR.**

| **Gene** | **Forward (5’ to 3’)** | **Reverse (5’ to 3’)** |
| --- | --- | --- |
| BMF | GCGCGGAGCCCTGGCATCAC | GCTGGTGTTGCTGCACATGAAGCCG |
| SOSTDC1 | CACTCGGGTTCAAGTGGGTT | GGCTCTTTTCCGCTCTCTGT |
| POU2F3 | TGGAGCCAGGAAATGATCGAAA | CCGCTTTGTTGCTGTGGAAA |
| LZTFL1 | AGCTGTGGTTCATAGTGAGGTG | TCCAGTGCATTTGTAGCCTGT |
| TMBIM1 | CGTGTCCTATGCTGTCTTCGT | TGATGGTGTGCTTCCGGTTC |
| ICAM1 | ACGGGAGCCAGCTGTGGGGG | CGTTGGGCGCCGGAAAGCTGTA |
| LIF | TGCAGCAGGGGTTTGTGGAG | CATGCCAGGTCAGACGCACA |
| GAPDH | GTCTCCTCTGACTTCAACAGCG | ACCACCCTGTTGCTGTAGCCAA |
| B2M | CCACTGAAAAAGATGAGTATGCCT | CCAATCCAAATGCGGCATCTTCA |
